# Risk factors for severe bacterial infections in patients with systemic autoimmune diseases receiving rituximab

**DOI:** 10.1007/s10067-014-2509-2

**Published:** 2014-02-02

**Authors:** Marion Heusele, Pierre Clerson, Benoit Guery, Marc Lambert, David Launay, Guillaume Lefevre, Sandrine Morell-Dubois, Hélène Maillard, Noémie Le Gouellec, Pierre-Yves Hatron, Eric Hachulla

**Affiliations:** 1 National Reference Centre for Systemic Autoimmune Diseases, Department of Internal Medicine, Claude Huriez Hospital, Université de Lille Nord-de-France, Lille, France; 2 Orgamétrie Biostatistiques Roubaix, Roubaix, France; 3 Infectious Diseases Department, Claude Huriez Hospital, Pavillon Fourrier, Université de Lille Nord-de-France, Lille, France; 4 Department of Internal Medicine, Hôpital Claude Huriez, 59037 Lille, France

**Keywords:** Infectious risk, Rituximab, Systemic autoimmune diseases, Systemic lupus erythematosus, Vasculitis

## Abstract

The risk of serious bacterial infectious events (SIEs) after an RTX course used in severe and refractory cases of systemic autoimmune diseases (SAID) is well known. Risk factors for SIEs merit investigation. For this case–control study, data were collected in a single centre of internal medicine and included all patients who received rituximab (RTX) for SAID between 2005 and 2011 (rheumatoid arthritis was excluded). Sixty-nine patients with SAID received a total of 87 RTX courses. Thirteen SIEs were reported in 12 patients leading to death in 5 patients. Patients with a history of SIE were significantly older (63.6 ± 18.8 vs 48.8 ± 16.7; *p* = 0.0091), suffered most frequently of diabetes mellitus (33.3 % vs 5.3 %, *p* = 0.015), had a lower CD19 count (1.0 ± 1.2/mm^3^ vs 3.9 ± 7.2/mm^3^) and had most frequently a prednisone dose >15 mg/day (91.7 % vs 47.7 %) at the start of the first RTX course. The SIE rate was 18.7 per 100 patient-years. At the initiation of the RTX course, risk factors for SIEs were lower IgG levels (OR = 0.87, 95%CI = 0.77–0.99, *p* = 0.03), lower CD19 count (OR = 0.85, 95%CI = 0.73–1.00) and creatinine clearance ≤ 45 ml/min (OR = 7.78, 95%CI = 1.36–44.38, *p* = 0.002). Conversely history of pneumococcal vaccination significantly decreased the risk of SIEs (OR = 0.11, 95%CI = 0.03–0.41, *p* = 0.0009). Concomitant treatment with prednisone at a dose >15 mg/day significantly increased the SIE risk (OR = 8.07, 95%CI = 1.94–33.59, *p* = 0.0004). SIEs are frequent in SAID treated with RTX, particularly in patients receiving high-dose corticosteroids, in patients with renal insufficiency and in patients with low IgG levels or a low CD19 count.

## Introduction

Biologic agents are regularly used for the management of systemic autoimmune diseases (SAID) refractory to conventional therapy or dependent of high corticosteroid doses [1]. Rituximab (RTX) is a monoclonal antibody that targets CD20+ B cells. The CD20 antigen is restricted to B lymphocytes at the pre-B cell stage but is not present in mature plasma cells or stem cells [2]. Peripheral B cell depletion lasts for an average of 6–9 months and sometimes longer [3]. B cells that return from the bone marrow to the peripheral blood after depletion are immature or naive, rather than memory B cells [4]. This delayed development of memory cells appears to persist for several years after RTX treatment [5].

Despite two negative randomized trials in systemic lupus erythematosus (SLE) [6, 7], RTX is frequently used in severe and refractory SLE. The positive results of two randomized clinical trials in antineutrophil cytoplasmic antibody (ANCA)-associated vasculitis [8, 9] will increase their use in this indication, sometimes as a first line therapy. The increased risk of infection due to RTX use in SAID has already been reported upon [10, 11]. The risk of serious infectious events (SIEs) remains several months after an RTX course, the majority within the 6 months of the RTX infusion [12]. The main goal of the study was to identify the risk factors for the SIEs occurring within 12 months after the first RTX administration in any course of RTX.

## Materials and methods

### Method

The primary objective of our study was to evaluate the risk factors of SIEs in RTX treated patients. This is a case–control study. Cases for analysis were defined as “RTX courses with occurrence of SIEs” and controls as “RTX courses without SIEs”. Data were collected in a single centre of internal medicine (National Centre for Rare Systemic Auto-immune Diseases, Lille, France). All patients who received off-label RTX for SAID between 2005 and 2011 were included in the study. Due to the individual and nominative dispensation of this drug, the central pharmaceutical department was able to forward an exhaustive list of all the patients treated in our department. There were no exclusion criteria; all patients were taken into account. A course of RTX was defined as IV infusions of either 2 × 1,000 mg given 2 weeks apart or 4 × 375 mg/m^2^ given 4 weeks apart. At each RTX infusion, 100-mg methylprednisolone was systematically administered intravenously to prevent perfusion reactions. SIEs were defined as any infection which led to hospitalization and/or death and/or required treatment with intravenous antibiotic/antiviral drugs. Nosocomial infections were defined as localized or systemic conditions that resulted from adverse reactions to the presence of (an) infectious agent(s) or its toxin(s) and that were not present or incubating at the time of hospital admission. For most bacterial nosocomial infections, this means that the infection usually becomes evident 48 h or more after admission [13]. History of hospitalization of at least 48 h within the previous 90 days [14] was also collected to individualize a subset of patients corresponding to health care-associated infections [15].

This research was authorized by the French competent authority dealing with research on human biological samples namely the French Ministry of Research. The authorization number is DC 2008 642. To issue such authorization, the Ministry of Research has sought the advice of an independent ethics committee, namely the “Comité de Protection des Personnes”. The committee voted positively on the quality of information provided to patients and the obtainment of consent for the intended use in research. Therefore, we attest that this study was conducted in accordance with French regulations concerning human research, and no breach to clinical research rules was observed.

### Statistics

SIEs were right censored at 12 months after the first perfusion of RTX for each treatment course. The influence of patient’s characteristics at the start of an RTX course was estimated by logistic regression models. Some patients underwent several courses of RTX, and, as analysis had focus on each RTX course and not on each patient, these RTX courses for a same patient could not be assumed to be independent. Therefore, the analysis was adjusted for clustered data (procedure SURVEYLOGISTIC). Results are expressed as odds ratios with their two-sided 95 % confidence intervals. Analyses were conducted using the SAS 9.1.3 statistical software (SAS Institute, Cary, NC, USA).

## Results

### Patients

Sixty-nine patients entered the study, and none were lost to follow-up. Among them, 55 patients received one course of treatment, 10 received two courses and 4 received three courses. A total of 87 RTX courses involving 69 patients were analysed. Fifty-six patients (81.2 %) were female; the mean age was 51.4 ± 18.1 years. Patients were suffering from SLE (*n* = 22), Sjögren’s syndrome-associated vasculitis (*n* = 14), ANCA-associated vasculitis (*n* = 9), idiopathic mixed cryoglobulinaemia vasculitis (*n* = 10), haematologic autoimmune disorders (autoimmune haemolytic anaemia/idiopathic thrombocytopenic purpura/acquired haemophilia with anti-factor VIII antibodies) (*n* = 12), myositis (*n* = 3) or catastrophic antiphospholipid syndrome (*n* = 1). RTX was mostly given to patients considered to be refractory to conventional therapy including corticosteroids and at least one immunosuppressive drug (*n* = 64, 92.8 %) or to patients who were dependent on a high dose of corticosteroid (i.e. prednisone 20 mg or more per day) for the purpose of corticosteroid sparing (*n* = 5, 7.2 %). Forty-three (62.3 %) patients had received pneumococcal vaccination (40 before and 3 after the first RTX course). Twelve patients experienced at least one SIE during or after an RTX course; one patient experienced two SIEs after the first and the third cure, respectively. Characteristics of patients with or without SIE before the first cure are given in Table 1. Comparisons involved 12 patients with at least one SIE and 57 patients who never had SIE. Patients with a history of SIE were significantly older, suffered most frequently of diabetes mellitus, had a lower CD19 count and had most frequently a prednisone dose >15 mg/day at the start of the first RTX course.

**Table 1:** Characteristics of patients with or without history of severe infection events

Status of the patients at the start of the first rituximab course	Patients with history of SIEs (*n* = 12)	Patients without history of SIEs (*n* = 57)	*p*
Mean age +/− SD	63.6 ± 18.8	48.8 ± 16.7	0.0091
Gender: male/female	3 (25 %)/9	10 (17.5 %)/47	0.68
SLE	3 (25 %)	19 (33.3 %)	0.74
Sjögren	1 (8.3 %)	13 (22.8 %)	0.44
ANCA vasculitis	2 (16.7)	7 (12.3 %)	0.65
Idiopathic cryoglobulinaemia vasculitis	4 (33.3 %)	6 (10.5 %)	0.06
Haematologic autoimmune disorders	4 (33.3 %)	8 (14.0 %)	0.20
Diabetes mellitus	4 (33.3 %)	3 (5.3 %)	0.015
Gammaglobulin level (g/l)	7.9 ± 5.4	11.4 ± 5.8	0.18
IgG level (g/l)	7.5 ± 4.1	11.9 ± 7.3	0.20
Lymphocyte count (100/mm^3^)	656.5 ± 597.1	1,353.1 ± 1,057.5	0.20
CD19 count (/mm^3^)	1.0 ± 1.2	3.9 ± 7.2	0.03
CD4 count (100/mm^3^)	550.1 ± 551.4	605.9 ± 465.6	0.78
Creatinine clearance (ml/min)	75.9 ± 41.4	85.4 ± 31.2	0.37
Creatinine clearance ≤45 ml/min	4 (33.3 %)	3 (5.3 %)	0.15
Creatinine clearance ≤60 ml/min	5 (41.7 %)	13 (22.8 %)	0.28
Concomitant immunosuppressive drug ^a^	2 (16.7 %)	20 (35.1 %)	0.31
Previous immunosuppressive drug ^a^	3 (25 %)	23 (40.4 %)	0.51
Prednisone >15 mg/day	11 (91.7 %)	27 (47.4 %)	0.005

^a^methotrexate (*N* = 9), azathioprine (*N* = 8), leflunomide (*N* = 4), mycophenolate mofetil (*N* = 4), cyclophosphamide (*N* = 1)

### RTX courses

Mean number of RTX perfusions was 2.9 ± 1.1 perfusions per RTX course. In patients who received several RTX courses, the mean delay between two courses was 36.6 ± 19.2 months. Immediate tolerability was good in all patients. Side effects were mild and common: diarrhoea (*n* = 2), headaches (*n* = 3), hyperthermia (*n* = 3), asthenia (*n* = 3), flu-like symptoms (*n* = 2) and mild upper respiratory tract infections (*n* = 11). One patient developed a serum sickness with arthralgia, cutaneous rash and hyperthermia 8 days after the end of the second course with a rapid spontaneous favourable evolution despite the first course having been well tolerated. During RTX courses, concomitant treatments were immunosuppressive drugs (*n* = 26, 29.9 %) and prednisone at a dose >15 mg/day (*n* = 41, 47.1 %). These treatments were pursued for a further 6 months after the end of the RTX course.

### Laboratory monitoring

Ig levels, CD4 lymphocyte count and CD19 lymphocyte count were monitored. Due to the observational nature of the study, data were not collected at regular intervals. Before the first RTX infusion for each cure, the mean IgG level was 9.9 ± 6.9 g/l (*n* = 51), and lymphocyte counts were 1,267 ± 976/mm^3^ with 627 ± 474 CD4/mm^3^ and 3.4 ± 6.4 CD19/mm^3^. During the 6 following months, the mean IgG level decreased to a nadir of 8.7 ± 4.8 g/mm^3^ (*n* = 56) compared to IgA or IgM which both remained virtually unchanged. Sixteen out of 42 patients with documented IgG values experienced an IgG level below 6 g/l, and ten had a nadir below 5 g/l. Post course CD19 counts were documented in 59 patients, and 31 (52.5 %) had no CD19 (i.e. a count equal to 0/mm^3^) after the RTX course. CD4 count decreased from 627 ± 474/mm^3^ to a nadir of 469 ± 384/mm^3^. In 14 out of 58 documented patients, the nadir was <200/mm^3^.

### Severe infections

Twelve patients experienced at least one SIE during or after an RTX course (17.4 % of patients). Thirteen SIEs were reported (14.9 % of RTX courses). All were bacterial or suspected bacterial infections. Within 6 months following the first infusion of a course, we observed 11 SIEs in 11 patients (12.6 % of RTX courses). Two other SIEs (one pneumonia and one sinusitis that occurred, respectively, 208 days and 345 days after the first RTX infusion of the course) occurred more than 6 months after the RTX course. Baseline characteristics of cases and controls at beginning of the RTX course are given in Table 2. Severe infection rate was 18.7 per 100 patient-years. Individual data of SIEs are provided in Table 3. However, if data are analysed as a percentage of the numbers of patients in each group, some particularities seem to emerge: 3 lupus patients out of 22 developed an SIE (13 %) in contrast to 2 out of 9 ANCA vasculitis (22 %), 4 out of 12 with a haematologic autoimmune disorder (33 %) and 4 out of 10 with idiopathic cryoglobulinaemia vasculitis (40 %). Over the 12-month period following the start of RTX course, five patients died from infections (5/12), and one died due to another cause (Paget’s disease of the breast at month 6). None of those who died from infection had received pneumococcal vaccination, and all had received prednisone at a dose >15 mg/day concomitant with RTX. Four of the five infected deceased patients were older than 70 years old.

**Table 2:** Individual data of patients with severe infections

	Sex/age	Diagnosis	CT and IS at time of SIE	PV	Infection/time from the RTX course	Pathogen	Outcome
1	F/45	V/SG	*P* > 15 mg/day	Y	Bronchitis/month 2	–	Favourable
2	M/72	V/CG	*P* > 15 mg/day	N	Febrile neutropenia/month 1	*Streptococcus pneumoniae*	Death Toxic shock syndrome
3	F/49	SLE	*P* < 15 mg/day	Y	Pneumonia/month 6	–	Favourable
4	F/73	SLE	*P* < 15 mg/day	N	Hand cellulitis/month 3	*Staphylococcus aureus methy-R*	Favourable
5	F/75	AH	*P* > 15 mg/day	N	Pneumonia/month 1	*Pseudomonas aeruginosa nombreux Pneumocystis + Candida albicans + Enterobacter cloacae* (nosocomial infection)	Death related to infection
6	F/75	V/CG	*P* > 15 mg/day	N	Suspected endocarditis/month 1	–	Death related to acute renal failure and infection
7	M/66	AH	*P* > 15 mg/day	Y	Septicemia/month 2	*Enterococcus faecalis* (possible nosocomial infection)	Favourable
8	F/21	SLE	IS + *P* > 15 mg/day	N	Pneumonia/month 1	– (possible nosocomial infection)	Favourable
9	F/90	AH	*P* > 15 mg/day	N	Febrile neutropenia/month 2	*Escherichia coli* (nosocomial infection)	Favourable
10	F/55	W	*P* > 15 mg/day	N	Pneumonia/month 1	*Enterococcus faecalis + Clostridium clostridiform + Bacterium lentum + Staphylococcus epidermidis + Stenotrophomonas maltrophilia* (nosocomial infection)	Death related to infection
11	F/77	AIHA	*P* > 15 mg/day	N	Post-surgical pneumonia/month 1	– (nosocomial infection)	Death following lung surgery in a septic context
12	F/47	SLE	*P* > 15 mg/day	N	Sinusitis/month 11	*Streptococcus pneumoniae PSDP* + *Haemophilus influenzae*	Favourable
13	M/70	W	*P* > 15 mg/day	N	Pneumonia/month 10	*Streptococcus pneumoniae* + *Haemophilus influenzae*	Favourable

*V* vasculitis, *SG* Sjögren syndrome, *CG* cryoglobulinaemia, *SLE* systemic lupus erythematosus, *AH* acquired haemophilia, *W* Wegener’s disease, *AIHA* autoimmune haemolytic anaemia, *RTX* rituximab, *PV* pneumococcal vaccination, *D* death, *Y* yes, *N* no, *CT* concomitant treatments, *P* prednisone, *IS* immunosuppressive drug

**Table 3:** Risk factors of baseline characteristics regarding the risk of severe bacterial infection (univariate analysis)

	OR^a^	95%CI	*P*
Age (years)	1.05	[0.99–1.10]	0.08
Female sex	0.58	[0.13–2.61]	0.48
Treatment with IVIg within 3 months prior to RTX course	1.74	[0.31–9.75]	0.53
Immunosuppressive drugs within 6 months before RTX course	0.82	[0.24–2.84]	0.75
Concomitant treatment with immunosuppressive drugs	0.38	[0.07–1.97]	0.25
High-dose IV methylprednisolone within 1 month before RTX course	2.17	[0.54–8.71]	0.28
Concomitant treatment with prednisone >15 mg/day	8.07	[1.94–33.59]	0.004
Statin use	1.92	[0.42–8.76]	0.40
Creatinine clearance >60 ml/min	0.51	[0.14–1.85]	0.31
Creatinine clearance ≤60 ml/min	1.94	[0.54–7.00]	0.31
Creatinine clearance ≤45 ml/min	7.78	[1.36–44.38]	0.02
Hospitalisation >48 h within 180 days before RTX course	1.94	[0.55–6.87]	0.30
Pneumococcal vaccination	0.11	[0.03–0.41]	0.0009
Nadir of gammaglobulin rate during the 6 months after RTX course (g/l)	0.67	[0.52–0.86]	0.002
IgG level at start of RTX course (g/l)	0.87	[0.77–0.99]	0.03
Lymphocyte count at start of RTX course (100/mm^3^)	0.83	[0.64–1.08]	0.17
CD19 count at start of RTX course (/mm^3^)	0.85	[0.73–1.00]	0.05
CD4 count at start of RTX course (100/mm^3^)	0.96	[0.76–1.21	0.71

*OR* odds ratio, *RTX* rituximab

^a^13 cases and 74 controls; OR provided by univariate regression models

*Streptococcus pneumoniae* was identified in 3/13 SIEs, one febrile neutropenia and sepsis that occurred within 1 month after the first dose of RTX course and one sinusitis and one pneumonia having occurred, respectively, 208 and 345 days after the beginning of the RTX course. For these two patients, the relationship with RTX was considered as probable as IgG levels were <6 g/l (respectively, 5.9 g/l and 1.79 g/l) at the time of infection. Six out of the 11 SIEs observed during the 6 months following the RTX course were nosocomial infections (occurring during a hospitalization period or during the 2 following days).

### Factors associated with SIEs occurring within 12 months following RTX courses

Risk factors for SIEs were investigated by logistic regression models for clustered data (Table 3). At the initiation of the RTX course, the risk factors for SIEs were lower IgG levels (OR = 0.87, 95%CI = 0.77–0.99, *p* = 0.03), lower CD19 count (OR = 0.85, 95%CI = 0.73–1.00) and creatinine clearance ≤45 ml/min (OR = 7.78 95%CI = 1.36–44.38, *p* = 0.002). Conversely, history of pneumococcal vaccination significantly decreased the risk of SIEs (OR = 0.11, 95%CI = 0.03–0.41, *p* = 0.0009). Concomitant treatment with prednisone at a dose >15 mg/day significantly increased the SIE risk (OR = 8.07, 95%CI = 1.94–33.59, *p* = 0.0004). We did not find a cutoff point either for IgG level (IgG <6 g/l: OR = 2.19, 95%CI = 0.52–9.22, *p* = 0.28) nor for gamma globulins level (gamma globulins <6 g/l: OR = 1.77 95%CI = 0.55–5.74, *p* = 0.34). Odds ratio for age at the time of RTX course did not reach statistical significance (*p* = 0.08) most probably partly due to the small number of events.

## Discussion

The main goal of this case–control study was to investigate risk factors for SIE in patients who received off-label RTX for SAID. Within 12 months following the first infusion of each RTX course, we observed 13 SIEs in 12 patients most of them occurring within the 2 months after the first perfusion of RTX. We found that patients with a history of SIE were significantly older, suffered most frequently of diabetes mellitus, had a lower CD19 count and had most frequently a prednisone dose >5 mg/day at the start of the first RTX course. Moreover, a lower level in gamma globulins, a lower CD19 count and renal failure (creatinine clearance ≤45 ml/min) at initiation of an RTX course increased the risk of SIE. Conversely, the SIE risk was lower in patients having been vaccinated against *S. pneumonia*. A concomitant treatment with prednisone at a dose >15 mg/day was associated with a higher risk of SIEs.

### Severe infectious event rate

SAIDs are at high risk of SIEs with a rate of 18.7 SIEs/100 patient-years. Other studies conducted in rheumatoid arthritis (RA) patients and SLE patients have found lower rates (around 4 to 6 SIEs/100 patient-years) [16, 17]. Diaz Lagarez et al. [10], however, found an 11.2 SIEs/100 patient-years SIE rate in patients suffering from SAID with a 22.6 SIEs/100 patient-years SIE rate in patients treated with more than two RTX courses. The risk of SIEs was not increased by addition of RTX to methotrexate (MTX) when compared to MTX alone. In the randomized EXPLORER study (phase II/III extra renal SLE evaluation of RTX), the SIE rate was lower in the RTX group compared to the placebo group (9.5 % and 17 %, respectively) [7]. Another randomized study [9] compared RTX and cyclophosphamide in patients with ANCA-associated renal vasculitis. There were 19 SIEs in 12 of the 33 patients treated by RTX (36 %) and 7 SIEs in 3 of the 11 patients (27 %) treated with cyclophosphamide. The difference was not significant between the two groups. Three patients died from infection in the RTX group versus one in the cyclophosphamide group. Van Vollenhoven et al. [18] studied the influence of the number of courses on the infection rate in RA patients treated with RTX and MTX. It emerged that neither the number of courses nor the total exposure time to RTX had any effect on the risk of SIEs. In our study, interestingly, patients with SLE seem at far less risk of developing SIEs. Indeed, the fact that the average age of patients with a history of infections is 63 years supports the view that SLE seems to be relatively at low risk for infection (given the fact that most lupus patients are much younger).

### Pathogen

No viral infection was observed in this cohort. We highlighted that, in 11 patients who had an SIE during the 6 months following the course, 2 had community pathogens, and 4 other patients had hospital-related pathogens. Six SIEs occurred in patients with a past history of an at least 48-h hospitalization within the 90 days before the SIE, suggesting that the SIE could be nosocomial. Similarly, Gottenberg et al. found pathogens frequently identified as possibly nosocomial and included *Pseudomonas aeruginosa*, *Escherichia coli*, and *Staphylococcus aureus* [17]. Thus, there is an increased risk of nosocomial infection which calls for caution in the use of RTX in the context of a previous prolonged hospitalization.

### Pneumococcal vaccination

Of the patients who developed SIEs, 72.7 % had not received pneumococcal vaccine before or during the RTX course versus 28.9 % of the patients who did not develop SIEs. In the subgroup of patients who had not received pneumococcal vaccine before the RTX course, one had severe pneumococcal sepsis and died, and two others had documented pneumococcal disease, albeit after the 6-month period following the RTX course. In the subgroup of vaccinated patients, one had received a pneumococcal vaccination 8 months after the first course (therefore, 3 months before a pneumococcal infection (patient 12)). The lymphocytic depletion was still complete at the time of the vaccination (CD19 number = 0/mm^3^), probably explaining the lack of efficacy of the vaccination. This highlights the importance of pneumococcal vaccination before the first RTX course (at least 3 to 4 weeks when possible) as recommended [19] and also the need to update all other non-live attenuated vaccines. The high number of pneumococcal infections has modified our practice. We now recommend pneumococcal vaccination in all SAID patients 3 to 4 weeks before the first course of RTX, when possible. If not, we propose pneumococcal vaccination simultaneously with the first RTX administration although it is known that the immune response against pneumococcal vaccination is reduced in RTX-treated patients even when the vaccine is administrated 28 weeks after the RTX course [3].

### Basal IgG level

Despite a lack of data concerning the IgG levels, it emerged that a high IgG level at baseline was associated with a lower risk of SIEs. Common observations showed that IgG’s are the most important Ig for protective immunity and that patients who have a low IgG level have an increased risk of SIEs. However, in the Van Vollenhoven et al. study [18], the effect of IgG at baseline was not significant. We advise discussion on the use of IVIg in patients who have an IgG level <5 g/l before RTX treatment, particularly in patients who have a past history of severe infection.

### Corticosteroid dosage

In 81.8 % of RTX courses with SIEs, the patients were concomitantly receiving prednisone at a dose >15 mg/day versus 42.1 % of RTX courses without SIE. In a meta-analysis focusing on the infectious risk in patients taking corticosteroids, no SIEs occurred when the prednisone daily dose was lower than 10 mg [20]. When possible, corticosteroids should be used at low doses in association with RTX. In lupus nephritis for example, it has previously been shown that remission is possible with immunosuppressive drugs and corticosteroids at a dose of 10 mg/day [21].

### Limitations

The major limitation of the study is its retrospective nature. Nevertheless, due to the traceability of RTX dispensation, all SAIDs treated in our department during the 2005–2011 period were collected, and no patients were lost to follow-up. The lack of a control group of patients suffering from SAID not exposed to RTX definitely makes it difficult to attribute the high rate of SIEs to the RTX use. Due to the multiple aspects of SAIDs, we cannot rule out that the type of SAID may influence the SIE risk. Multivariate analysis was not possible due to the small number of events. Finally, due to the low number of patients involved who were retreated, it is not possible to evaluate the effect of retreatment on the risk of SIEs.

## Conclusions

This study identified four main risk factors associated with an increased risk of SIEs in SAID patients treated with RTX: high-dose corticosteroids (>15 mg/day), renal insufficiency (creatinine clearance ≤45 ml/min), a low IgG level and a low CD19 count. The high number of pneumococcal infections has modified our practice. We would recommend pneumococcal vaccination in all SAID patients 3 to 4 weeks prior to the first course of RTX if possible or simultaneously to the first RTX course if not possible in case of an emergency situation. IVIg could be discussed in patients who have an IgG level <5 g/l, particularly if SIEs occur, though this deserves further studies. Last but not least, we recommend, when possible, to limit the corticosteroid dose below 15 mg/day or to rapidly decrease the corticosteroid dose below 15 mg/day after induction treatment in SAID treated with RTX.
